# biojs-io-biom, a BioJS component for handling data in Biological Observation Matrix (BIOM) format

**DOI:** 10.12688/f1000research.9618.2

**Published:** 2017-01-09

**Authors:** Markus J. Ankenbrand, Niklas Terhoeven, Sonja Hohlfeld, Frank Förster, Alexander Keller

**Affiliations:** 1 Department of Animal Ecology and Tropical Biology (Zoology III), University of Würzburg, Würzburg, Germany; 2 Department of Plant Physiology and Biophysics (Botany I), University of Würzburg, Würzburg, Germany; 3 Center for Computational and Theoretical Biology (CCTB), University of Würzburg, Würzburg, Germany; 4 Department of Bioinformatics, University of Würzburg, Würzburg, Germany

**Keywords:** biom-format, ecology, meta-genomics, biojs, parser, meta-barcoding

## Abstract

The Biological Observation Matrix (BIOM) format is widely used to store data from high-throughput studies. It aims at increasing interoperability of bioinformatic tools that process this data. However, due to multiple versions and implementation details, working with this format can be tricky. Currently, libraries in Python, R and Perl are available, whilst such for JavaScript are lacking. Here, we present a BioJS component for parsing BIOM data in all format versions. It supports import, modification, and export via a unified interface. This module aims to facilitate the development of web applications that use BIOM data. Finally, we demonstrate its usefulness by two applications that already use this component.

**Availability:** https://github.com/molbiodiv/biojs-io-biom, https://dx.doi.org/10.5281/zenodo.218277

## Introduction

In recent years, there has been an enormous increase in biological data available from high-throughput studies. Complications arise from the enlarged size of the resulting data tables. This is the case for transcriptomic and marker-gene community data, where the central matrix consists of counts for each observation (e.g. gene or taxon) in each sample, plus a second and third matrix for metadata of both taxa and samples, respectively.

Early on there have been efforts to define data formats that capture all relevant information for an experiment like the Minimum Information About a Microarray Experiment (MIAME) project ^1^. In 2005 the Genomic Standards Consortium (GSC) formed with the mission of enabling genomic data integration, discovery and comparison through international community-driven standards ^2^. The Biological Observation Matrix (BIOM) Format was developed to standardize the storage of observation counts together with all relevant metadata and it is a member project of the GSC ^3^. One main purpose of the BIOM format is to enhance interoperability between different software suits. Many current leading tools in community ecology and metagenomics support the BIOM format, e.g. QIIME ^4^, MG-RAST ^5^, PICRUSt ^6^, phyloseq ^7^, VAMPS ^8^ and Phinch ^9^. Additionally, libraries exist in Python ^3^, R ^10^ and Perl ^11^ to propagate the standardized use of the format.

Interactive visualization of biological data in a web browser is becoming more and more popular ^12, 13^. For the development of web applications that support BIOM data, a corresponding library is currently lacking and would be very useful, since several challenges arise when trying to handle BIOM data. While BIOM format version 1.0 builds on the JSON format and thus is natively supported by JavaScript, the more recent BIOM format version 2.1 uses HDF5 and can therefore not be handled natively in web browsers. Also the internal data storage can be either dense or sparse so applications have to handle both cases. Furthermore application developers need to be very careful when modifying BIOM data as changes that do not abide to the specification will break interoperability with other tools. Here we present biojs-io-biom, a JavaScript module that provides a unified interface to read, modify, and write BIOM data. It can be readily used as a library by applications that need to handle BIOM data for import or export directly in the browser. To demonstrate the utility of our module it has been used to implement a simple user interface for the biom-conversion-server ^14^. Additionally, the popular BIOM visualization tool Phinch ^9^ has been extended with new features, in particular support for BIOM version 2.1 by integrating biojs-io-biom ^15^.

## The biojs-io-biom component

The biojs-io-biom library can be used to create new objects (called Biom objects for brevity) by either loading file content directly via the static parse function or by initialization with a JSON object:



var biom = new Biom({
    id: ’My Biom’,
    matrix_type: ’dense’,
    shape: [2,2],
    rows: [
        {id: ’row1’, metadata: {}},
        {id: ’row2’, metadata: {}}
    ],
    columns: [
        {id: ’col1’, metadata: {}},
        {id: ’col2’, metadata: {}}
	],
    data: [
        [0,1],
        [2,3]
    ]
});
                


The data is checked for integrity and compliance with the BIOM specification. Missing fields are created with default content. All operations that set attributes of the Biom object with the dot notation are also checked and prompt an error if they are not allowed.



                    var biom = new Biom({});
biom.id = [];
// Will throw a TypeError as id has to be a string or null
                


Beside checking and maintaining integrity the biojs-io-biom library implements convenience functions. This includes getter and setter for metadata as well as data accessor functions that are agnostic to internal representation (dense or sparse). But one of the main features of this library is the capability of handling BIOM data in both versions 1.0 and 2.1 by interfacing with the biom-conversion-server ^14^. Handling of BIOM version 2.1 in JavaScript directly is not possible due to its HDF5 binary format. The only reference implementation of the format is in C and trying to transpile the library to JavaScript using emscripten ^16^ failed due to strong reliance on file operations (see discussions in ^17, 18^). Using the conversion server allows developers to use BIOM of both versions transparently. Biom objects also expose the function write which exports it as version 1.0 or version 2.1. In contrast to the existing biom_convert module for the Galaxy platform which has a rich set of options the biom-conversion-server exhibits its functionality both via an API and a simple user interface that does not need any kind of setup or login ^19, 20^.

## Application

To demonstrate the utility of this module it has been used to implement a user interface for the biom-conversion-server ^14^. Besides providing an API it is now also possible to upload files using a file dialog. The uploaded file is checked using our module and converted to version 1.0 on the fly if necessary. It can then be downloaded in both version 1.0 and 2.1. As most of the functionality is provided by the biojs-io-biom module the whole interface is simply implemented with a few additional lines of code.

As a second example the Phinch framework ^9^ has been enhanced to allow BIOM version 2.1. Phinch visualizes the content of BIOM files using a variety of interactive plots. However due to the difficulties of handling HDF5 data only BIOM version 1.0 is supported. This is unfortunate as most tools nowadays return BIOM version 2.1 (e.g. QIIME from version 1.9,1 ^4^ and Qiita ^21^). It is possible to convert from version 2.1 to version 1.0 without loss of information but that requires an extra step using the command line. By including our biojs-io-biom module and the biom-conversion-server into Phinch it was possible to add support for BIOM version 2.1 along with some other improvements ^15^.

As the biojs-io-biom module resolves the import and export challenges, one of the next steps is the development of a further BioJS module to present BIOM data as a set of data tables. In order to do that for large datasets sophisticated, accessor functions capitalizing on the sparse data representation have to be implemented.

A drawback of the internal storage of BIOM version 1.0 is that it suffers of those shortcomings that are solved in version 2.1, specifically efficient handling of huge datasets. However even with a more efficient data storage huge amounts of data will still cause problems with current web browsers. Therefore, we plan on extending the biom-conversion-server with a light communication API that allows a client to request only the subsets of the full data set that it requires.

## Conclusion

The module biojs-io-biom was developed to enhance the import and export of BIOM data into JavaScript. Its utility and versatility has been demonstrated in two example applications. It is implemented using latest web technologies, well tested and well documented. It provides a unified interface and abstracts from details like version or internal data representation. Therefore, it will facilitate the development of web applications that rely on the BIOM format.

## Software availability

### biojs-io-biom

Latest source code https://github.com/molbiodiv/biojs-io-biom

Archived source code as at the time of publication https://zenodo.org/record/218277

License MIT

### biom-conversion-server

Latest source code https://github.com/molbiodiv/biom-conversion-server

Archived source code as at the time of publication https://zenodo.org/record/218396

Public instance https://biomcs.iimog.org

License MIT
