# Supplementary material for: biojs-io-biom, a BioJS component for handling data in Biological Observation Matrix (BIOM) format
Source: F1000Res. 2017 Jan 9;5:2348. Originally published 2016 Sep 20. [Version 2] doi: 10.12688/f1000research.9618.2 (PMC5224677; doi:10.12688/f1000research.9618.2)
Supplement: Peer review history

# Peer review: Referee response for version 2

**DOI:** 10.5256/f1000research.11389.r19078

**Published:** 2018-04-19

**Authors:** Holly M. Bik

**Affiliations:** 1 Department of Nematology, University of California Riverside, Riverside, CA, USA

The authors have made significant changes and the revised manuscript is much improved over the original version. The focus on the biojs-io-biom tool provides a much better rationale and manuscript structure. I am satisfied with the author responses to my previous comments and now happy to give final approval to this manuscript.

I have read this submission. I believe that I have an appropriate level of expertise to confirm that it is of an acceptable scientific standard.

# Peer review: Referee response for version 2

**DOI:** 10.5256/f1000research.11389.r19077

**Published:** 2017-01-09

**Authors:** Joseph Nathaniel Paulson

**Affiliations:** 1 Department of Biostatistics, Harvard T.H. Chan School of Public Health, Boston, MA, USA

The authors addressed my main concerns and I have noticed that the documentation is much better on the github page. Good job

I have read this submission. I believe that I have an appropriate level of expertise to confirm that it is of an acceptable scientific standard.

# Peer review: Referee response for version 1

**DOI:** 10.5256/f1000research.10362.r16545

**Published:** 2016-10-25

**Authors:** Joseph Nathaniel Paulson

**Affiliations:** 1 Department of Biostatistics, Harvard T.H. Chan School of Public Health, Boston, MA, USA

Ankenbrand *et al.* provide a javascript library to interact with the microbial consortia BIOM format version 1 class. As the authors note, a javascript library could be a great benefit to the community as many commonly used tools like QIIME and Mothur produce BIOM formatted objects. However, the article and software are missing a few key components for a fully positive review.

 Major comments:

 There is a historical context that Ankenbrand *et al.* miss in discussing biom-format and subsequently imply that the biom-format is more widely adopted than being field specific format. If the authors leave the introduction more general, then I would suggest they include more background on the history of high-throughput data storage and reproducibility in programmatic languages, perhaps starting with the Minimum Information About a Microarray Experiment - MIAME format ^1^ and exprSet classes developed in R about 15 years ago before the genomics standards consortium (formed in 2005), for which biom-format is a member.

 The authors posit that the BIOM format version 2 / 2.1 that moved to HDF5 made it impossible for javascript libraries to manipulate it natively. We found a javascript library that “takes advantage of the compatibility of V8 and HDF5”. Were the authors unable to build from this library to take advantage of the version 2 BIOM format? The BIOM version 2 / 2.1 formats were designed specifically to handle many of the shortcomings of the version 1 in terms of memory and design. It would be advantageous of the users to build from this if possible to at least read in the BIOM v2.1 HDF5 files.

 In my own installation of the software, I keep getting error messages when I attempt to create a biom object, see here: http://tinyurl.com/f1000-review. If the reviewers could please clarify the installation guide on the github repo.

 Minor comments:

 The second sentence needs clarification. “Despite this increase, for many of these studies the general basic layout of the data is similar to traditional assessment after bioinformatical processing, yet complications arise due to the increased size of the data tables.”

 The citation for the BIOM interface R package has been deprecated. The appropriate citation is: Paul J. McMurdie and Joseph N Paulson (2015). *biomformat: An interface package for the BIOM file format.* R/Bioconductor package version 1.0.0. ^2^.

I have read this submission. I believe that I have an appropriate level of expertise to confirm that it is of an acceptable scientific standard, however I have significant reservations, as outlined above.

## References

[1] BrazmaAHingampPQuackenbushJSherlockGSpellmanPStoeckertCAachJAnsorgeWBallCACaustonHCGaasterlandTGlenissonPHolstegeFCKimIFMarkowitzVMateseJCParkinsonHRobinsonASarkansUSchulze-KremerSStewartJTaylorRViloJVingronM: Minimum information about a microarray experiment (MIAME)-toward standards for microarray data. *Nat Genet*.2001;29(4) : 10.1038/ng1201-365 365-71 10.1038/ng1201-365 11726920

[2] McMurdiePJPaulsonJN: biomformat: An interface package for the BIOM file format. *R/Bioconductor package version 1.0.0*.2015; Reference source

# Response

**Published:** 2016-12-23

**Authors:** Markus J. Ankenbrand

**Affiliations:** University of Würzburg, Germany

Thanks a lot for the thorough review and the good suggestions for improvement. Find our point by point answers below (original comments in bold):

**There is a historical context that Ankenbrand et al. miss in discussing biom-format and subsequently imply that the biom-format is more widely adopted than being field specific format. If the authors leave the introduction more general, then I would suggest they include more background on the history of high-throughput data storage and reproducibility in programmatic languages, perhaps starting with the Minimum Information About a Microarray Experiment - MIAME format 1 and exprSet classes developed in R about 15 years ago before the genomics standards consortium (formed in 2005), for which biom-format is a member.**

 As suggested we extended the introduction to cover more of the historical context.

**The authors posit that the BIOM format version 2 / 2.1 that moved to HDF5 made it impossible for javascript libraries to manipulate it natively. We found a javascript library that “takes advantage of the compatibility of V8 and HDF5”. Were the authors unable to build from this library to take advantage of the version 2 BIOM format? The BIOM version 2 / 2.1 formats were designed specifically to handle many of the shortcomings of the version 1 in terms of memory and design. It would be advantageous of the users to build from this if possible to at least read in the BIOM v2.1 HDF5 files.**

 There is a fine distinction between JavaScript inside a browser and on a server (nodejs) that we previously did not make sufficiently clear in our manuscript. For the nodejs environment there is in fact a library that handles data in HDF5 format (https://github.com/HDF-NI/hdf5.node). As our library is supposed to work equally well in both environments we tried to port this library to the browser. Unfortunately that proofed to be infeasible even after contacting the developers of the library (see https://github.com/HDF-NI/hdf5.node/issues/29). We adjusted the manuscript to make clear that HDF5 is not natively supported in the browser rather than in javascript in general. Further we added a section discussing the downside of being limited to JSON and plans to overcome that at the end of the Application section.

**In my own installation of the software, I keep getting error messages when I attempt to create a biom object, see here: http://tinyurl.com/f1000-review. If the reviewers could please clarify the installation guide on the github repo.**

 Thanks for finding that issue. We fixed the bug creating your issue, added a minimum required version of nodejs and improved the documentation.

**The second sentence needs clarification. “Despite this increase, for many of these studies the general basic layout of the data is similar to traditional assessment after bioinformatical processing, yet complications arise due to the increased size of the data tables.”**

 Rephrased

**The citation for the BIOM interface R package has been deprecated. The appropriate citation is: Paul J. McMurdie and Joseph N Paulson (2015). biomformat: An interface package for the BIOM file format. R/Bioconductor package version 1.0.0.2.**

 Fixed

# Peer review: Referee response for version 1

**DOI:** 10.5256/f1000research.10362.r16436

**Published:** 2016-10-18

**Authors:** Holly M. Bik

**Affiliations:** 1 Department of Nematology, University of California Riverside, Riverside, CA, USA

This manuscript describes the biojs-io-biom toolkit, which includes a conversion library and server for re-formatting Biological Observation Matrix (BIOM) files between versions 1.x (JSON-formatted) and 2.x (HDF5-formatted).

 The conversion library itself is extremely useful, since it will allow users to convert quickly between BIOM file formats without having to go back to the command line (e.g. QIIME) and easily reformat files for use in various applications.

 I do not have the necessary javascript expertise to comment on the codebase and conversion server backend, so I will offer some general comments on the practical applications outlined in the text:

 Since this project is based on the Phinch framework, I find the "Blackbird" rebranding of the fork to be very problematic. The "Blackbird" instance is really just an updated release of the Phinch framework, with some bug fixes, added features, and implementation of the new BIOM conversion server. The rebranding/renaming is confusing for the end user (see comment by other peer reviewer below), and mistakenly implies a number of scenarios that are not accurate: 1) that the authors were involved in the original development of data visualization tools, 2) that the Blackbird rebranding and design changes were approved from by the original developers, and 3) the "Blackbird" project represents a significant expansion or retooling of the current Phinch framework. I’m fully aware that this is open source software and the authors are free to reuse and share the Phinch codebase, but I don't really see the utility of the "Blackbird" rebranding, and creating an additional web instance that mostly replicates the functionality of http://phinch.org will confuse end users.

 Since the authors here are really community contributors to the original Phinch project, I would recommend eliminating the "Blackbird" rebranding of the project, and reverting back to Phinch branding (citing the framework release as Phinch v2.0). We will then initiate a pull request to update the bug fixes and integrate the new biojs-io-biom source code to be live on http://phinch.org  The visual layout for Phinch (name, logo and visualization layout) was thoughtfully constructed, and the new Blackbird logo and visual modifications will likely interfere with “brand recognition” that should be attributed to the original Phinch framework.

 Once this pull request is initiated and completed, the “Application” manuscript text should be updated to reflect the live implementation of the conversion library on a v2.0 Phinch framework at phinch.org.

 Other minor comments:

- Can you please provide details on how and where the "Blackbird" instance and biom-conversion-server are currently hosted (e.g. Amazon AWS)?
- Please list the public landing page for the applications mentioned in the text (in case users want to access these tools directly) - e.g. https://biomcs.iimog.org
- The biom-conversion-server does not appear to be backwards compatible (I could not upload and convert a BIOM 1.x file to 2.x format) - this one-way conversion functionality is should be clearly indicated in the first paragraph of the “Application” section. In addition, if users try to upload a BIOM 1.0 file they should be presented with an appropriate error message (I didn’t see one - the tool just froze when I attempted to upload a BIOM 1.0 file).
- There are other BIOM conversion servers that exist, e.g. implementations within the Galaxy framework - see https://toolshed.g2.bx.psu.edu/repository/display_tool?repository_id=b3ae8ca9317b000e&render_repository_actions_for=tool_shed&tool_config=%2Fsrv%2Ftoolshed%2Fmain%2Fvar%2Fdata%2Frepos%2F002%2Frepo_2436%2Fbiom_convert.xml&changeset_revision=501c21cce614 - these alternate tools should be mentioned in the text. How does the biom-conversion-server compare with (and potentially improve on) such Galaxy based tools?

I have read this submission. I believe that I have an appropriate level of expertise to confirm that it is of an acceptable scientific standard, however I have significant reservations, as outlined above.

# Response

**Published:** 2016-12-23

**Authors:** Markus J. Ankenbrand

**Affiliations:** University of Würzburg, Germany

Thanks a lot for taking the time to review this article and for the good suggestions for improvement. Find our point by point answers below (original comments in bold):

**Since this project is based on the Phinch framework, I find the "Blackbird" rebranding of the fork to be very problematic. The "Blackbird" instance is really just an updated release of the Phinch framework, with some bug fixes, added features, and implementation of the new BIOM conversion server. The rebranding/renaming is confusing for the end user (see comment by other peer reviewer below), and mistakenly implies a number of scenarios that are not accurate:**

**1) that the authors were involved in the original development of data visualization tools,**

**2) that the Blackbird rebranding and design changes were approved from by the original developers, and**

**3) the "Blackbird" project represents a significant expansion or retooling of the current Phinch framework.**

**I’m fully aware that this is open source software and the authors are free to reuse and share the Phinch codebase, but I don't really see the utility of the "Blackbird" rebranding, and creating an additional web instance that mostly replicates the functionality of http://phinch.org will confuse end users. Since the authors here are really community contributors to the original Phinch project, I would recommend eliminating the "Blackbird" rebranding of the project, and reverting back to Phinch branding (citing the framework release as Phinch v2.0).We will then initiate a pull request to update the bug fixes and integrate the new biojs-io-biom source code to be live on http://phinch.org The visual layout for Phinch (name, logo and visualization layout) was thoughtfully constructed, and the new Blackbird logo and visual modifications will likely interfere with “brand recognition” that should be attributed to the original Phinch framework. Once this pull request is initiated and completed, the “Application” manuscript text should be updated to reflect the live implementation of the conversion library on a v2.0 Phinch framework at phinch.org.**

 Thanks for sharing your thoughts on this delicate topic. We are grateful to you for suggesting a more satisfactory solution. As you suggested we prepared the pull request that integrates the additional features into Phinch and removed Blackbird branding from our fork. We look forward to the changes going live on phinch.org. We will use the same procedure for future improvements as long as you are interested in merging them.

**Can you please provide details on how and where the "Blackbird" instance and biom-conversion-server are currently hosted (e.g. Amazon AWS)?**

 The biom-conversion-server and the Phinch preview instance are both docker containers currently running on a virtual machine with Ubuntu 16.04 (2GB RAM, 1CPU) on a dedicated server hosted by Hetzner.

**Please list the public landing page for the applications mentioned in the text (in case users want to access these tools directly) - e.g. https://biomcs.iimog.org**

 Added links to the manuscript

**The biom-conversion-server does not appear to be backwards compatible (I could not upload and convert a BIOM 1.x file to 2.x format) - this one-way conversion functionality is should be clearly indicated in the first paragraph of the “Application” section. In addition, if users try to upload a BIOM 1.0 file they should be presented with an appropriate error message (I didn’t see one - the tool just froze when I attempted to upload a BIOM 1.0 file).**

 In general the biom-conversion-server is not limited to one way conversion. Attempts to replicate the described behaviour were not successful so it might be a problem with a specific BIOM file. We are eager to find the cause of this issue and opened a bug report here: https://github.com/molbiodiv/biom-conversion-server/issues/4

 However we need your assistance in tracking down this bug.

**There are other BIOM conversion servers that exist, e.g. implementations within the Galaxy framework - see https://toolshed.g2.bx.psu.edu/repository/display_tool?repository_id=b3ae8ca9317b000e&render_repository_actions_for=tool_shed&tool_config=%2Fsrv%2Ftoolshed%2Fmain%2Fvar%2Fdata%2Frepos%2F002%2Frepo_2436%2Fbiom_convert.xml&changeset_revision=501c21cce614 - these alternate tools should be mentioned in the text. How does the biom-conversion-server compare with (and potentially improve on) such Galaxy based tools?**

 Thanks for pointing that out. We included the Galaxy biom_convert tool in our discussion.

# Peer review: Referee response for version 1

**DOI:** 10.5256/f1000research.10362.r16546

**Published:** 2016-10-03

**Authors:** Daniel McDonald, Evan Bolyen

**Affiliations:** 1 Department of Pediatrics, University of California, San Diego, La Jolla, CA, USA; 2 Center for Microbial Genetics and Genomics, Northern Arizona University, Flagstaff, AZ, USA

In Ankenbrand *et al*, the authors develop a library to enable interaction with BIOM, a file format common in the microbiome field, from the JavaScript programming language. JavaScript is a staple of web-development, and the ability to interact with BIOM formatted files via JavaScript will facilitate the development of web-based tools for microbiome research. As the authors note, libraries for the interaction BIOM files have only been implemented so far in Python, R and Perl. And while Python and Perl have a strong web presence, they are not natively supported in modern web browsers as JavaScript is, and often rely on server-side processing as opposed to the client-side paradigms which JavaScript excels at.

**General comments**

- The API provided by BioJS is minimal. Notably, methods for partitioning, collapsing, transforming, filtering and subsampling are not present. While developers will be able to access sample or observation profiles as a whole, the current release of BioJS pushes much of the common manipulation logic onto the consumer of the library.
- The in memory representation of the data following parse by BioJS are either in a dense matrix, or in a dict of keys style sparse representation. As the authors note, specialized methods will need to be created to handle large data efficiently, however the authors may wish to consider placing emphasis instead on specialized data structures such as compressed sparse row or column.
- The highlight with Blackbird is great to see but we were confused by the intention of the Github fork. The codebase suggests that it is more than just a proof of concept to highlight BioJS as there is project-specific branding. Would the authors consider clarifying their position with Blackbird?
- The primary motivator for the development of BIOM-format 2.1.0 were scaling limitations inherent with the JSON-based representation of 1.0.0. Specifically, the “data” key of the JSON string must be parsed in full in order to random access to individual sample or observation data. This removes the possibility of algorithms which depend on efficient random access patterns for data too large for main memory. Additionally, the overhead associated with representing a large JSON object in memory is high. While we acknowledge HDF5 possesses challenges for web-based interaction with these data, it is important to note that the 1.0.0 JSON-based format is not recommended for modern sized studies using hundreds to thousands to tens of thousands of samples.
- The use of the conversion server is very cool and could be taken a step further by layering a light communication API on top to allow a client to request arbitrary samples. This separation would remove the burden of the client needing to read HDF5 formatted files, greatly lower the memory footprint of the client, and likely be more performant than a pure client-side model as the client would only need to know about what it had requested. This expansion of biojs-io-biom, in our opinion, would have the greatest impact for expanding the use of BIOM formatted data within a web application.

**Major**

- When the authors refer to BIOM v2, we believe they are actually referring to BIOM v2.1.0. There are important distinctions between the format versions. Would the authors consider clarifying the minor version number in discussion?

**Minor**

- The two uses of “accession functions” reads awkwardly as these types of methods are generally described as “accessor functions.” Would the authors consider revising the phrasing?

**Disclosures**

 Daniel McDonald and Evan Bolyen are developers for the BIOM-Format Project.

We have read this submission. We believe that we have an appropriate level of expertise to confirm that it is of an acceptable scientific standard, however we have significant reservations, as outlined above.

# Response

**Published:** 2016-12-23

**Authors:** Markus J. Ankenbrand

**Affiliations:** University of Würzburg, Germany

We thank the reviewers for their constructive comments that helped us improve the manuscript. Find our point by point answers below (original comments in bold):

**The API provided by BioJS is minimal. Notably, methods for partitioning, collapsing, transforming, filtering and subsampling are not present. While developers will be able to access sample or observation profiles as a whole, the current release of BioJS pushes much of the common manipulation logic onto the consumer of the library.**

 Thanks for pointing that out. We continuously add more functions to make use of our library more convenient. I opened a dedicated issue listing the functions that are present in the python library but lacking in ours (https://github.com/molbiodiv/biojs-io-biom/issues/16). We already implemented functions for transformation, normalization and filtering in order to get more feature complete.

**The in memory representation of the data following parse by BioJS are either in a dense matrix, or in a dict of keys style sparse representation. As the authors note, specialized methods will need to be created to handle large data efficiently, however the authors may wish to consider placing emphasis instead on specialized data structures such as compressed sparse row or column.**

 That is a very good point and something we are evaluating at the moment.

**The highlight with Blackbird is great to see but we were confused by the intention of the Github fork. The codebase suggests that it is more than just a proof of concept to highlight BioJS as there is project-specific branding. Would the authors consider clarifying their position with Blackbird?**

 After feedback from Holly Bik (Principal Investigator on the Phinch framework) we agreed to remove the Blackbird branding and instead merge our improvements back into Phinch. Therefore, we removed references to Blackbird from the manuscript. For more details see the referee report by Holly Bik (18 Oct 2016) and this discussion on GitHub: https://github.com/PitchInteractiveInc/Phinch/issues/63

**The primary motivator for the development of BIOM-format 2.1.0 were scaling limitations inherent with the JSON-based representation of 1.0.0. Specifically, the “data” key of the JSON string must be parsed in full in order to random access to individual sample or observation data. This removes the possibility of algorithms which depend on efficient random access patterns for data too large for main memory. Additionally, the overhead associated with representing a large JSON object in memory is high. While we acknowledge HDF5 possesses challenges for web-based interaction with these data, it is important to note that the 1.0.0 JSON-based format is not recommended for modern sized studies using hundreds to thousands to tens of thousands of samples.**

 This is a valid point. By using the JSON representation for our library we re-introduce the limitations of BIOM-format 1.0. We hope to support the HDF5 format in the future. However even with support of HDF5 loading full tables with tens of thousands of samples into the browser might be too memory intensive. Therefore, the next thing we would like to try is the extension of the conversion server with the communication API as you suggested. We added a short paragraph clearly stating our shortcoming and discussing the possible solution at the end of the Application section.

**The use of the conversion server is very cool and could be taken a step further by layering a light communication API on top to allow a client to request arbitrary samples. This separation would remove the burden of the client needing to read HDF5 formatted files, greatly lower the memory footprint of the client, and likely be more performant than a pure client-side model as the client would only need to know about what it had requested. This expansion of biojs-io-biom, in our opinion, would have the greatest impact for expanding the use of BIOM formatted data within a web application.**

 This is a great suggestion and we are eager to work on that for the next major release. We also added this as a future prospect to the manuscript.

**When the authors refer to BIOM v2, we believe they are actually referring to BIOM v2.1.0. There are important distinctions between the format versions. Would the authors consider clarifying the minor version number in discussion?**

 We added the minor version number whenever we refer to the BIOM format. We left the patch level out as the documentation on biom-format.org only lists the three versions (1.0, 2.0, 2.1). If you feel that the patch level is relevant as well we will gladly add that, too.

**The two uses of “accession functions” reads awkwardly as these types of methods are generally described as “accessor functions.” Would the authors consider revising the phrasing?**

 Thanks a lot. We revised the phrasing.
